# Cost‐effectiveness of an app‐based treatment for urinary incontinence in comparison with care‐as‐usual in Dutch general practice: a pragmatic randomised controlled trial over 12 months

**DOI:** 10.1111/1471-0528.17191

**Published:** 2022-05-31

**Authors:** Anne M. M. Loohuis, Henk Van Der Worp, Nienke J. Wessels, Janny H. Dekker, Marijke C. Ph. Slieker‐Ten Hove, Marjolein Y. Berger, Karin M. Vermeulen, Marco H. Blanker

**Affiliations:** ^1^ Department of General Practice and Elderly Care Medicine University Medical Centre Groningen, University of Groningen Groningen The Netherlands; ^2^ Profundum Institute, Education and Research Dordrecht The Netherlands; ^3^ Department of Epidemiology University Medical Centre Groningen, University of Groningen Groningen The Netherlands

**Keywords:** app, cost‐effectiveness, eHealth, general practice, long‐term, pragmatic, primary care, self‐management, urinary incontinence

## Abstract

**Objective:**

To assess the cost‐effectiveness of app‐based treatment for female stress, urgency or mixed urinary incontinence (UI) compared with care‐as‐usual in Dutch primary care.

**Design:**

A pragmatic, randomised controlled, superiority trial.

**Setting:**

Primary care in the Netherlands from 2015 to 2018, follow‐up at 12 months.

**Population:**

Women with ≥2 UI‐episodes per week, access to mobile apps, wanting treatment.

**Methods:**

The standalone app included conservative management for UI with motivation aids (e.g. reminders). Care‐as‐usual delivered according to the Dutch GP guideline for UI.

**Main outcome measures:**

Costs and cost‐effectiveness and ‐utility were assessed from a societal perspective, based on incontinence impact adjusted life years (IIALYs), quality adjusted life years (QALYs) and medical, non‐medical and productivity costs. Information on costs was obtained with the iMCQ and iPCQ questionnaires (medical consumption and productivity cost questionnaires).

**Results:**

In all, 262 women were andomised equally to app or care‐as‐usual; 89 (68%) and 83 (63%) attended follow‐up, respectively. Costs were lower for app‐based treatment with € −161 (95% confidence interval [CI −180 to −151) per year. Cost‐effectiveness showed small mean differences in effect for IIALY (0.04) and QALY (−0.03) and thus larger incremental cost‐effectiveness ratios (ICER: −€3696) and incremental cost‐utility ratios (ICUR: €6379).

**Conclusion:**

App‐based treatment is a cost‐effective alternative to care‐as‐usual for women with UI in Dutch primary care.

**Tweetable abstract:**

App‐treatment for female urinary incontinence cost‐effective compared to care‐as‐usual in general practice after 12 months.

## Tweetable abstract

App‐treatment for female urinary incontinence cost‐effective compared to care‐as‐usual in general practice after 12 months.

Linked article: See related article at https://doi.org/10.1111/1471‐0528.16875.

## 1 INTRODUCTION

With one in three women being affected, urinary incontinence (UI) has a large impact on woman. This is accompanied by substantial costs. We have recently shown that an eHealth application for the treatment of UI is effective, in both the short‐ and long‐term, as described in this issue.^1^, ^2^ Although this suggests that app treatment can be provided as an alternative, we postulate that before a larger implementation of this eHealth treatment, insight into costs is of importance. In two recent Swedish trials, the effectiveness and cost‐effectiveness of an internet‐based programme and mobile app for treating stress UI were studied.^3^, ^4^, ^5^, ^6^ For this, researchers compared the interventions with postponed treatment or a postal‐based programme.^3^, ^4^, ^5^, ^6^ Those studies did not include women with urgency UI or mixed UI. So, the cost‐effectiveness of an eHealth application for all common types of UI have not been compared with care‐as‐usual. In the current study, we aimed to assess the costs as well as cost‐effectiveness of our app‐based treatment compared with care‐as‐usual by Dutch GPs.

## 2 METHODS

### 2.1 Study design

We performed a pragmatic, parallel arm, randomised controlled trial to compare app‐based treatment and care‐as‐usual in a general practice setting, for women with stress, urgency or mixed UI. The study design, recruitment challenges and primary outcome (non‐inferiority of treatment after 4 months) have been published in detail.^1^, ^7^, ^8^ Elsewhere in this issue, we reported the long‐term effectiveness.^2^ In this report, we focus on the secondary analysis of the cost‐effectiveness after 12 months.

From July 2015 through July 2018, we recruited adult Dutch women with stress, urgency or mixed UI via general practices, the lay press and social media. See Appendix S1 for the full inclusion and exclusion criteria. Data collection included a physical and urogynaecological examination at baseline,^9^ web‐based questionnaires, and a 3‐day frequency‐volume chart at baseline and after 4 and 12 months.

### 2.2 Randomisation and blinding

One of two researchers (AMML and NJW, both GP trainee) confirmed eligibility, gained signed informed consent, and collected baseline data. After enrolment of the participant in the study, women were randomised using the computer programme ALEA, which allowed full concealment of group allocation.^10^ Participants were randomised with 1:1 allocation and random block sizes stratified at the GP level^7^ Treatment allocation could not be blinded to participants and caregivers due to the study design.

### 2.3 Interventions

Women in the intervention group gained access to the URinControl App, which was based on relevant guidelines for treating UI.^11^, ^12^ Women in the care‐as‐usual group were referred to their own GP to discuss treatment options. GPs were advised to follow the Dutch GP guideline on UI. No limitations on the type and mode of treatment were applied.^11^ Both interventions are outlined in detail in Appendix S1.

### 2.4 Outcomes

Costs were measured at a patient level at both 4 and 12 months based on enquiries about medical and non‐medical consumption and productivity over the past 4 months. We used the adapted medical consumption and productivity cost questionnaires (iMCQ and iPCQ) from the Institute of Medical Technology Assessment and included the costs of app development and maintenance. We doubled the costs measured at 12 months to estimate costs between 4 and 12 months. We rated cost components collected during the trial based on the standard Dutch guideline for economic evaluations composed by the Dutch National Health Care Institute.^13^ The sum of costs was recorded as the total societal cost. All costs are presented in euros based on the 2017 year‐end prices (2014 prices indexed to inflation by 2.414%). Productivity losses included costs by absenteeism from work, due to any health problem. This was calculated by the friction‐cost method.^14^ We did not include the cost for an individual's time invested performing pelvic floor muscle training (PFMT) or bladder training because asking women to track this was considered too time‐consuming in relation to the relatively low anticipated costs. Yearly costs for app development and maintenance were based on the actual costs. A scenario of 30 000 users was used, derived as a conservative estimate from the number of users of freely available apps for UI and on the number of downloads of the Swedish Tät app.^15^

For the cost analysis, effectiveness was measured with the incontinence impact adjusted life years (IIALY) score derived from the ICIQ‐UI‐SF symptom score.^16^ The IIALY score reflects disease‐specific quality of life weighted from the patient's perspective with a score from 0 (severe impact of UI on quality of life) to 1 (no impact of UI on quality of life). Utility was based on the EQ‐5D‐5L, with valuations generated using the Dutch tariff for the EQ‐5D.^17^ The EQ‐5D questionnaire is a generic quality of life questionnaire that generates preference‐based scores from −0.33 (severe problems on all five dimensions) to 1 (best possible health state). Areas under the receiver operating characteristic curve were used to calculate the IIALYs and QALYs gained for each individual during the 12‐month follow‐up period: to gain one IIALY or one QALY at a population level (i.e. to add one additional life year in perfect health), the calculated amount (in euros) would need to be invested.

### 2.5 Statistical methods

We assessed effect on quality of life by linear regression on an intention to treat basis, with results considered statistically significant if the *P*‐value was <0.05. We compared baseline characteristics of the final cohort with those of the group lost to follow‐up with linear regression and non‐parametric tests. Data were analysed with IBM SPSS version 26.0 (IBM Corp.) and R Studio version 1.2.5033.

The economic evaluation was conducted from a societal perspective, including direct and indirect medical and non‐medical costs over 12 months. Incremental costs per IIALY gained were expressed as an incremental cost‐effectiveness ratio (ICER). The balance between costs and QALYs were expressed as an incremental cost‐utility ratio (ICUR).^13^ Costs and effects were recorded and calculated on an individual basis and the mean differences between the two study groups were then calculated. The ICER and ICUR represent the average incremental cost needed to be invested to achieve 1 additional unit of the measure of effect and were computed by dividing the differences in mean effects and mean costs (as shown in Appendix S1). By performing 5000 bootstrap replications of the trial data, alternative confidence intervals were calculated based on the 2.5th and 97.5th centiles. Cost‐effectiveness planes visualise the uncertainty surrounding the ICER and ICUR. If cost‐effectiveness is implied based on the app‐based treatment saving costs and both treatments showing similar effects, it is not of added value to show the probability of this cost‐effectiveness (which is already implied) in an acceptability curve. Additionally, we performed a sensitivity analysis for a scenario with higher costs for app maintenance and extra costs for annual development. Data robustness was assessed using the mean of the follow‐up data at 4 and 12 months to estimate costs between 4 and 12 months instead of doubling the costs at 12 months. Finally, we performed subgroup analyses with the type of recruitment or type of UI.

## 3 RESULTS

**FIGURE 1:**
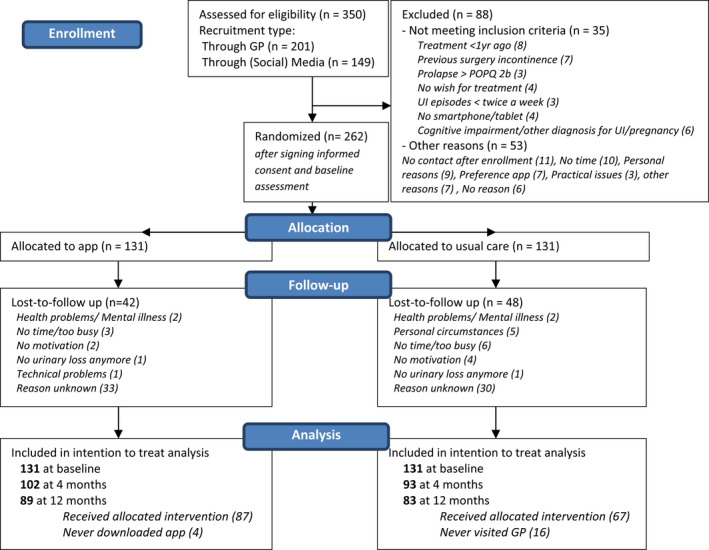
CONSORT flow diagram of participant recruitment. POPQ, pelvic organ prolapse quantification; UI, urinary incontinence. Reproduced with permission from Reference^2^.

Of 262 eligible women, 131 women were allocated to app‐based treatment, and 131 to care‐as‐usual (Figure 1). Baseline characteristics are presented in Table 1. Data from 89 women (68%) in the app‐based treatment group and 83 (63%) in the care‐as‐usual group were available for the intention‐to‐treat analysis. Despite differences in age and body mass index, we found no differences between participants with and without follow‐up data (Table S1). Details on the provided treatments are presented in Table S2.

**TABLE 1:** Baseline characteristics of women with complete follow‐up data shown by treatment group

Characteristics	App‐treatment	*n* ^a^	Care‐as‐usual	*n* ^a^
Age, (years)	54.9 ± 12.2	89	52.0 ± 9.8	83
Higher educational level	43 (51.8%)	83	40 (50.6%)	79
Body mass index (kg/m^2^)	26.6 ± 5.0	89	28.0 ± 5.4	83
Duration of UI (years)	8 (4–14)	89	8 (4–14)	83
Type of UI		89		83
Stress	34 (38.2%)		36 (43.4%)	
Mixed, stress predominant	24 (27.0%)		23 (27.7%)	
Urgency	9 (10.1%)		8 (9.6%)	
Mixed, urgency predominant	22 (24.7%)		16 (19.3%)	
Incontinence severity				
ICIQ‐UI SF score	9.2 ± 3.0	88	10.5 ± 3.1	83
ICIQ‐LUTSqol score	33.1 ± 7.5	88	33.4 ± 7.2	83
Generic quality of life score (EQ‐5D‐5L)	0.864 ± 0.19	88	0.896 ± 0.17	83
Makes use of incontinence products, yes	69 (80.2%)	86	68 (84.0%)	81
If yes, mean number of products per day	2 (1–4)	69	2 (1–3.75)	68
Previous treatment for UI		89		83
None	67 (75.3%)		58 (69.9%)	
Pessary	–		1 (1.2%)	
Physical therapist	22 (24.7%)		24 (28.9%)	

*Note:* Values are means ± standard deviation, numbers (%) or medians (interquartile range). Educational level was assessed at follow‐up.

Abbreviations: ICIQ‐LUTSqol, ICIQ lower urinary tract symptoms quality of life; ICIQ‐UI SF, International Consultation on Incontinence Modular Questionnaire Urinary Incontinence Short Form; UI, urinary incontinence.

^a^ *n* varied because of missing data of one baseline assessment and three baseline questionnaires. Reproduced with permission from Reference ^2^.

### 3.1 Effectiveness

Symptom severity, disease‐specific quality of life and generic quality of life scores at baseline, 4 and 12 months, and the change scores from baseline and adjusted differences between groups are presented in Table S3.

### 3.2 Costs

The mean direct and indirect cost per participant in the app‐based treatment group was €1520 (95% confidence interval [CI] 1512–1532), including €87 (95% CI 85–86) for UI‐specific costs. The mean direct and indirect cost per participant in the care‐as‐usual group was €1680 (95% CI 1673–1693), including €191 (95% CI 192–195) for UI‐specific costs (Table S4). For both the app‐based treatment and care‐as‐usual groups, incontinence material drove much of the UI‐specific costs (€62 and €80, respectively). Compared with app‐based treatment, care‐as‐usual was associated with higher costs for physical therapy, medication and other treatments for UI, equating to mean differences of €82, €9 and €8 per patient per year, respectively. The cost of app‐usage was €1.10 per patient per year based on the scenario of 30 000 users.

### 3.3 Cost‐effectiveness and cost‐utility analyses

**FIGURE 2:**
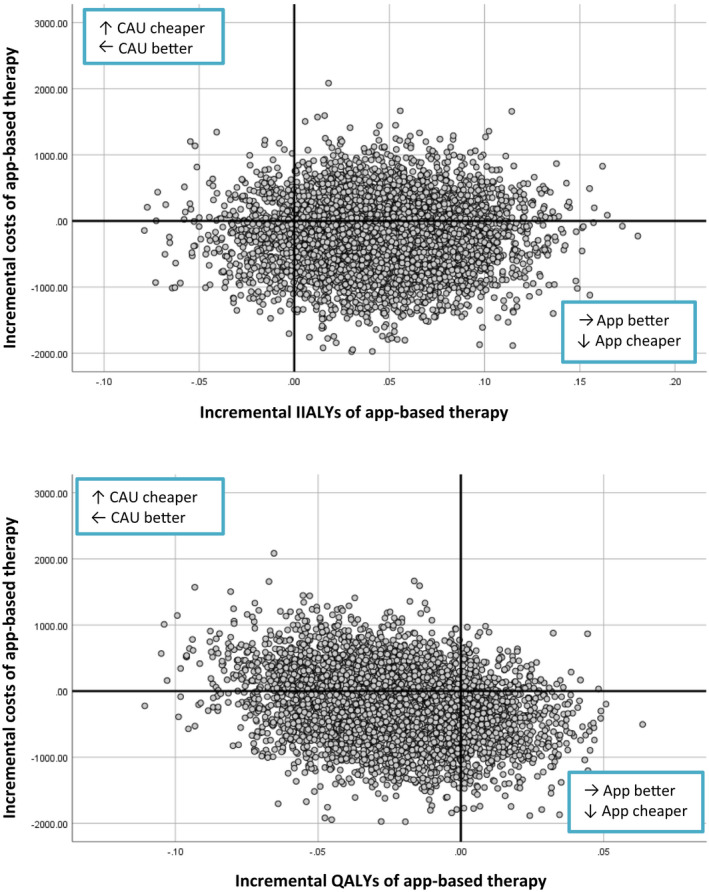
Incremental cost‐effectiveness planes per outcome parameter. CAU, care‐as‐usual; IIALY, incontinence impact adjusted life years; QALY, quality adjusted life years

The cost‐effectiveness analysis showed that the mean difference in effect gained per IIALY was 0.043 more for app‐based treatment than for care‐as‐usual. The mean difference in costs was €161 less (95% CI −180 to −151) in the app‐based treatment group, giving an ICER of ‐€3696 (95% CI −6716 to 12 712). The cost‐utility analysis revealed that there was a mean difference of −0.025 QALYs (i.e. fewer) for app‐based treatment than for care‐as‐usual, with an ICUR of €6379 (95% CI −4128 to 21 769) (Table 2, Figure 2).

**TABLE 2:** Cost‐effectiveness of app‐based treatment for urinary incontinence for women in general practice

	Treatment group		Mean difference	ICER (95% CI)
	App‐based	Care‐as‐usual		
	*n* = 87 ^a^	*n* = 82^a^		
IIALYs gained	0.71 ± 0.215	0.66 ± 0.250	0.043	€ −3696 (−6716 to 12 712)
Costs	1520 ± 3425	1680 ± 3357	−161	
				**ICUR (95% CI)**
QALYs gained	0.89 ± 0.165	0.91 ± 0.145	−0.025	€6379 (−4128 to 12 769)
Costs	1520 ± 3425	1680 ± 3357	−161	

Abbreviations: ICER, incremental cost‐effectiveness ratio; ICUR, incremental cost‐utility ratio; IIALYs, incontinence impact adjusted life years; QALYs, quality adjusted life years.

^a^ Three cases were excluded from the analyses because a large influence on the data due to outliers in costs.

In total, 65.6% of the 5000 replications in the bootstrap simulation were in the lower half of the plane, indicating lower costs for app‐based treatment (Figure 2). Moreover, any effects and utilities gained were comparable, with minimal differences between the groups in either IIALY (0.043) or QALY (−0.025) (Table 2).

### 3.4 Sensitivity and subgroup analyses

App‐based treatment remained cost‐effective when assessed with fewer app users, extra developmental and higher maintenance costs (Table S5). Sensitivity analysis using the mean costs at the 4 and 12 month follow‐up revealed comparable results, demonstrating the robustness of the cost calculation.

Subgroup analysis revealed differences in effects and costs by UI type and recruitment type (Table S6). App‐based treatment for urgency UI resulted in higher IIALYs gained (0.74) compared with care‐as‐usual (0.60). The costs for UI‐specific treatment were also approximately €60 higher for urgency UI compared with stress UI mainly due to the cost of incontinence material. Subgroup analysis by recruitment type showed that, for care‐as‐usual, the group recruited through (social) media had lower costs (€131) and a lower treatment effect (IIALY 0.64) than the group recruited by a GP (€235, IIALY 0.68). These cost differences were mainly based on lower use of physical therapy (€56 versus €122) and other treatments (e.g. pessary or tension‐free vaginal tape: €2 versus €86).

## 4 DISCUSSION

### 4.1 Main findings

This study, conducted in Dutch general practice, showed that an app‐based treatment for female stress, urgency and mixed UI was a cost‐effective alternative to care‐as‐usual. The clinical outcomes of both treatments did not differ but the app‐based treatment was less expensive than care‐as‐usual, with mean differences of €161 and €87 per patient per year in total and UI‐specific costs, respectively. The gained effects and utilities were comparable between groups after 1 year, with only small mean differences in the IIALY (0.043) and the QALY (−0.025). This resulted in an ICER of –€3696 and an ICUR of €6379. These results were robust and remained valid in a scenario that included higher app development costs.

### 4.2 Strengths and limitations

The main strength of this study is that we compared app‐based treatment with care‐as‐usual for women with all types of UI. The pragmatic design is considered the gold standard for economic evaluations in healthcare.^18^ Other strengths include the use of patient‐centred and validated outcome measures, the 12‐month follow‐up period, and the inclusion of sensitivity analyses to confirm the robustness of our data.

The cost and effect analyses were sufficient to make valid conclusions about cost‐effectiveness. The ICER and ICUR are typically used to represent costs associated with 1 unit of health gain. In our study, health gains for both treatments were comparable. The differences between treatments were minimal (EQ‐5D‐5L: 0.025, IIALY: 0.043) and were not statistically significant or clinically relevant.^19^ These minimal differences in effect resulted in high positive and negative ratios of ICER and ICUR. This may cause confusion because it would seem that these analyses are contradictory to each other. Therefore, we set the difference to focus on cost rather than health gains, given that the latter was comparable between the groups. Our rationale behind choosing this approach was aimed at being both pragmatic and informative.

Yearly costs for the app‐based treatment could only be based on assumptions, therefore we chose not to include this in the model as it might add uncertainty. Costs for an app‐based treatment result from initial development and annual maintenance, which were very low for the URinControl‐app, were €30,000 and €3,000, respectively.^20^ If we would use a scenario of 30 000 users, derived as a conservative estimate from the number of users of freely available apps for UI and on the number of downloads of the Swedish Tät app, costs would be just €1.10 per patient per year. For developmental costs, we could also imagine these are costs that can be regarded as the same type of costs as education of healthcare personnel. Maintenance costs, if applicable, are highly dependent on the app. For example, for the implementation phase of the URinControl‐app, there will be no maintenance costs, as the app is integrated in a larger eHealth‐platform.

Limitations that must be considered are power and loss to follow‐up. Often, cost‐effectiveness studies are underpowered because their power depends on the primary outcome measure of a trial. This trial was powered on non‐inferiority of effectiveness after 4 months. In this secondary analysis, 172 women (65.6%) were available for follow‐up and the power was lower. By performing a bootstrap analysis, this issue does not affect the results of the cost‐effectiveness analysis. However, the lower power must be considered in our effectiveness and subgroup analyses. Loss to follow‐up was associated with higher body mass index. Participation of these women could have further improved effects and lowered costs for both treatment groups, as weight loss is effective for UI and is a cheap intervention.^12^

### 4.3 Interpretation (in light of other evidence)

Our study findings are consistent with those from two other studies concluding that app‐ or internet‐based treatment is a cost‐effective alternative when managing UI.^5^, ^6^ These studies compared an app‐based approach with either a postal‐based programme or postponement of treatment, and assessed their cost‐effectiveness for stress UI in superiority trials. However, in any such evaluation, it is recommended to use a pragmatic design with a control group that reflects usual care.^18^ The current study is the first to conduct such a comparison. Our results indicate that app‐based treatment is a cost‐effective alternative for women with UI who present to general practice.

The UI‐specific follow‐up costs over 12 months in our data were comparable to other studies, but our total costs were higher for both app‐based treatment and care‐as‐usual (€1520 and €1680, respectively) compared with the data provided by Sjöström et al. comparing cost‐effectiveness of an app‐based treatment to no treatment (€547 and €482, respectively) and Vermeulen et al. comparing a pro‐active approach in diagnostic testing and treatment to usual care (€417 and €87, respectively).^6^, ^16^ Although these studies used a societal perspective, there were some differences in the cost analysis. Vermeulen et al. did not take into account productivity losses, as the mean age of their population was higher than retirement age, and Sjöström et al. mainly focused on the disease‐specific costs. We took into consideration a broader range of costs unrelated to UI to conduct the societal perspective as thoroughly as possible, as was advised by the Dutch guideline for economic evaluations composed by the Dutch National Health Care Institute.^13^

Although there are no UI‐specific references available, in the Netherlands the proposed values for cost utility are stratified by burden of disease (three categories). Assuming UI falls within the lowest burden of disease, a maximum value of €20,000 per QALY is advised. NICE in the UK used a more formal threshold of £20,000 –30,000 per QALY and in the US values range between $50,000 and $150,000 per QALY. Our estimates fall well within these boundaries.

Our subgroup analysis showed that app‐based treatment for urgency UI had higher treatment effects on the impact of incontinence on daily life (0.74 IIALYs) than did care‐as‐usual for urgency UI (0.60 IIALYs). This may be a result ofthe accessibility of the app, which helps distract women from feelings of urgency and to monitor the bladder training (e.g. the pee button). The treatment of urgency UI with an eHealth approach has not been studied before, precluding meaningful comparison.

## 5 CONCLUSION

### 5.1 Practical recommendations

Based on the short‐ and long‐term outcomes presented elsewhere and in this report, we believe app‐based treatment can be recommended as a viable alternative to care‐as‐usual in general practice.^1^, ^2^ This alternative will lower barriers to seeking and receiving help for UI for many women. We see two ways of promoting this application. First, GPs or physical therapists, specialised in pelvic floor dysfunctions, can offer the app to women who seek help for UI. Secondly, it can be promoted through (social) media and offered online, allowing it to reach women with UI that may not otherwise seek care but still have a latent treatment wish.^21^

### 5.2 Research recommendations

To ensure successful implementation and treatment efficacy, the final step needed is to identify the factors associated with treatment success and failure. Clarifying these factors could help to improve the app content and to ensure that it targets the most appropriate populations, including women with low literacy scales. Collecting user feedback and evaluating log data will be important to evaluate and improve the implementation process. Ultimately, women should be able to receive advice on the applicability of initiating app‐treatment in their personal situation.

We conclude that the app‐based treatment for stress, urgency and mixed female UI can be recommended as a cost‐effective alternative to care‐as‐usual in general practice.

## CONFLICT OF INTERESTS

None declared. Completed disclosure of interest forms are available to view online as supporting information.

## AUTHOR CONTRIBUTIONS

AMML collected the data, did the analysis, and wrote the paper. HVDW assisted with the analysis and contributed to the writing of the paper. NJW collected the data and contributed to writing the paper. JHD designed the study, acquired the funding, and contributed to writing the paper. MCSTH contributed to the study design, the app content, and the writing. MYB assisted in the study design and contributed to writing the paper. KMV assisted with the analysis and contributed to writing the paper. MHB designed the study, acquired the funding, was project leader, contributed to the analysis, contributed to writing the paper, and is the guarantor. The corresponding author attests that all listed authors meet the authorship criteria and that no others meeting the criteria have been omitted.

## ETHICS APPROVAL

The Medical Ethical Review Board of the University Medical Center Groningen, Netherlands, approved this study on 12‐05‐2015 (METc‐number: 2014/574). All participants gave written informed consent.

## Supporting information


Appendix S1

ICMJE

ICMJE

ICMJE

ICMJE

ICMJE

ICMJE

ICMJE

## Data Availability

Not applicable.
